# Broad Spectrum Antimicrobial Activity of Forest-Derived Soil Actinomycete, *Nocardia* sp. PB-52

**DOI:** 10.3389/fmicb.2016.00347

**Published:** 2016-03-18

**Authors:** Priyanka Sharma, Mohan C. Kalita, Debajit Thakur

**Affiliations:** ^1^Microbial Biotechnology Laboratory, Life Sciences Division, Institute of Advanced Study in Science and TechnologyGuwahati, India; ^2^Department of Biotechnology, Gauhati UniversityGuwahati, India

**Keywords:** *Nocardia* sp., antimicrobial activity, microbial pathogens, biosynthetic genes, culturing conditions, SEM, GC-MS

## Abstract

A mesophilic actinomycete strain designated as PB-52 was isolated from soil samples of Pobitora Wildlife Sanctuary of Assam, India. Based on phenotypic and molecular characteristics, the strain was identified as *Nocardia* sp. which shares 99.7% sequence similarity with *Nocardia niigatensis* IFM 0330 (NR_112195). The strain is a Gram-positive filamentous bacterium with rugose spore surface which exhibited a wide range of antimicrobial activity against Gram-positive bacteria including methicillin-resistant *Staphylococcus aureus* (MRSA), Gram-negative bacteria, and yeasts. Optimization for the growth and antimicrobial activity of the strain PB-52 was carried out in batch culture under shaking condition. The optimum growth and antimicrobial potential of the strain were recorded in GLM medium at 28°C, initial pH 7.4 of the medium and incubation period of 8 days. Based on polyketide synthases (PKS) and non-ribosomal peptide synthetases (NRPS) gene-targeted PCR amplification, the occurrence of both of these biosynthetic pathways was detected which might be involved in the production of antimicrobial compounds in PB-52. Extract of the fermented broth culture of PB-52 was prepared with organic solvent extraction method using ethyl acetate. The ethyl acetate extract of PB-52 (EA-PB-52) showed lowest minimum inhibitory concentration (MIC) against *S. aureus* MTCC 96 (0.975 μg/mL) whereas highest was recorded against *Klebsiella pneumoniae* ATCC 13883 (62.5 μg/mL). Scanning electron microscopy (SEM) revealed that treatment of the test microorganisms with EA-PB-52 destroyed the targeted cells with prominent loss of cell shape and integrity. In order to determine the constituents responsible for its antimicrobial activity, EA-PB-52 was subjected to chemical analysis using gas chromatography-mass spectrometry (GC-MS). GC-MS analysis showed the presence of twelve different chemical constituents in the extract, some of which are reported to possess diverse biological activity. These results confirmed that the presence of bioactive constituents in EA-PB-52 could be a promising source for the development of potent antimicrobial agents effective against wide range of microbial pathogens including MRSA.

## Introduction

The discovery of antibiotics like penicillin and other antimicrobial agents to treat infectious diseases has revolutionized the field of medicine in the mid-twentieth century. These discoveries have led to the development of improved antibiotics with a hope to serve humanity well (Walsh, 2003). However, due to overuse or misuse of antibiotics over a prolonged period, most of the pathogens have become resistant to the antibiotic therapy. These pathogens have accumulated a large number of resistance elements encoded by genes found both within the genome and plasmids, greatly limiting the therapeutic options (Wright, 2012). Thus, there is an immense need for the discovery and development of new antibiotics to effectively target these deadly pathogens that cause life-threatening infections. Actinomycetes are one of the most efficient groups of natural bioactive metabolite producers such as antibiotics, enzyme inhibitors, immunomodifiers, plant growth promoting substances, and many other compounds useful to mankind (Fiedler et al., 2004; Shivlata and Satyanarayana, 2015). Among actinomycetes, around 75% of commercially useful antibiotics such as ivermectin, tetracycline, streptomycin, nystatin etc. are produced by the dominant genus *Streptomyces* (Miao and Davies, 2010). But, in the past two decades, there has been a decline in the discovery of novel metabolites from *Streptomyces* as culture extracts usually yield disappointingly high number of previously described molecules (Qin et al., 2009; Aouiche et al., 2011). As such, new sources of bioactive metabolites from another group of actinomycetes, known as rare actinomycetes from different ecological niches have promoted recent advances in the discovery of new antibiotic molecules (Lazzarini et al., 2000; Lee et al., 2014; Azman et al., 2015; Nimaichand et al., 2015). The genus *Nocardia, Saccharopolyspora, Micromonospora, Streptosporangium, Streptoverticillium, Actinoplanes*, and *Actinomadura* are considered as the rare group of actinomycetes. It is because these microbes are difficult to isolate and maintain under conventional conditions (Berdy, 2005). Amongst the rare actinomycetes, numerous interesting biologically active compounds have been reported from the genus *Nocardia* such as nargenicin (Celmer et al., 1980), transvalencin (Hoshino et al., 2004), nocardithiocin (Mukai et al., 2009) etc.

*Nocardia* is a genus under the family Nocardiaceae of order Corynebacteriales within the class Actinomycetes (Goodfellow et al., 2012). The genus was first proposed by Trevisan (1889) and was named in honor of Edmond Nocard, who in 1888 described the first species (Kageyama et al., 2004a). *Nocardia* is a Gram-positive, aerobic, filamentous branching bacillus that is partially acid fast and ~86 species have been reported in the genus *Nocardia* (Brown-Elliott et al., 2015). It is represented by a list of chemical markers, including the presence of mycolic acids, meso-DAP, galactose, and arabinose and DNA G + C content of 63–72% (Goodfellow, 1992). However, little attention has been paid to *Nocardia* from where we continue our interest to extract biologically active compounds from this group of bacteria. They are prominent for their ability to produce a wide variety of biologically active compounds; however, some are also known to be opportunistically pathogenic to humans and animals (Chun and Goodfellow, 1995).

The plausibility of finding new bioactive molecules from *Nocardia* could be increased by shifting the search from routinely explored ecological niches to unexplored ones (Manikkam et al., 2014). The poorly explored environments contain highest populations of actinomycetes with valuable antimicrobial secondary metabolites as reported by Ara et al. (2007). Isolation of microorganisms from poorly explored areas of the world like Jordan (Saadoun and Gharaibeh, 2003), Antarctica (Lee et al., 2012), and certain ecological niches of Northeast India (Bordoloi et al., 2001; Debnath et al., 2013) reflects that these habitats should be carefully explored for novel microorganisms and their valuable bioactive products. Northeast India is a part of the Indo-Burma mega-biodiversity hotspot (Myers et al., 2000). The influence of the local environment in the biodiversity hotspots might result in the evolution of novel secondary metabolic pathways in organisms (Glover, 1995). Pobitora Wildlife Sanctuary of Assam is largely an unscreened forest ecosystem and thus an unexplored source of actinomycetes and biologically active secondary metabolites. Many actinomycetes from forest ecosystems are known to produce polyketides and non-ribosomal peptides by type-I and type-II polyketide pathways and non-ribosomal peptide synthase pathways which are the hallmark of secondary metabolites production in this group of bacteria (Passari et al., 2015).

Our continuous screening for new bioactive metabolites from rare actinomycetes resulted in the isolation of a promising *Nocardia* strain designated as PB-52 from the soil samples of Pobitora Wildlife Sanctuary of Assam, India. This sanctuary (38.8 sq km) lies in the sub-tropical zone and is situated in the flood plains of river Brahmaputra in Assam, India. In this work, we aimed to investigate the antimicrobial biosynthetic potential of PB-52 strain against a wide range of microbial pathogens. Optimization of different culture conditions like fermentation media, temperature, pH and period of incubation was performed to facilitate improved growth and antimicrobial activity of the strain. To evaluate the antimicrobial potency of ethyl acetate extract of PB-52 (EA-PB-52), various techniques like MIC determination against microbial pathogens, rate kill assay, interaction with the test microbial pathogens by SEM analysis was performed. The chemical constituents present in EA-PB-52 were further identified using GC-MS. The outcome of this research lays the foundation for performing in-depth studies focusing on the development of potent antimicrobial agents effective against a broad range of disease-causing microbial pathogens including methicillin-resistant *Staphylococcus aureus* (MRSA) and *Candida albicans*.

## Materials and methods

### Sampling site and sample collection

Soil samples were collected in the month of April, 2013 from varied locations of Pobitora Wildlife Sanctuary (26°12′ to 26°16′N and 91°58′ to 92°05′E) of Assam, India. The Sanctuary experiences semi-dry hot climate in summer (37–39°C) and cold in winter (6–7°C) with an average humidity of 75% and annual rainfall of 1500–2600 mm. The habitat comprises of alluvial grassland with hilly forests. Soil samples each weighing ~50 g was collected randomly from 5 to 20 cm depth after removing the upper surface layer of the top soil. The samples were transferred aseptically in sterile zip-lock bags and transported to the laboratory on the same day.

### Isolation of the actinomycete strain

During the screening of actinomycetes from soil samples, PB-52 strain was isolated by serial dilution technique in actinomycetes isolation agar medium (Himedia, India) amended with rifampicin (2.5 μg/mL) and amphotericin B (75 μg/mL) after incubation at 28°C for 7–10 days (Thakur et al., 2007). The pure culture of the isolate was sub-cultured on GLM agar medium (Yeast extract, 3 g; malt extract, 3 g; peptone Type I, 5 g; starch, 10 g; agar, 20 g; distilled water, 1000 mL; pH 7.4) and preserved in 15% glycerol at −80°C for future use.

### Identification and characterization of the actinomycete strain

#### Cultural characteristics

The cultural characteristic of PB-52 strain was examined by growing the strain in different culture media. Micromorphology of the strain was examined by using cover slip insertion method (Williams et al., 1989) on GLM medium. The morphology and ornamentation of the spore chain was observed by scanning electron microscopy (Kumar et al., 2011). Utilization of carbon and nitrogen was determined by growth of the strain on ISP Medium No. 9 supplemented with 1% carbon and 1% nitrogen sources respectively at 28°C. Temperature range (15–45°C), pH range (4–11) and NaCl tolerance for growth (1–5% NaCl, w/v) was determined on ISP Medium No. 4 (Shirling and Gottlieb, 1966). Hydrolysis of starch, cellulose, casein, tween 20, tween 80, liquefaction of gelatin, nitrate reduction and other biochemical tests were assessed by following the methods of Gordon et al. (1974). Whole-cell hydrolysates were determined according to Lechevalier and Lechevalier (1970). Sensitivity and resistance of PB-52 strain to nineteen standard antibiotics were detected by disc diffusion method (Kumar V. et al., 2014a).

### Molecular identification

#### DNA extraction

Genomic DNA was isolated according to Sambrook and Russell (2001) with slight modifications. For isolation of genomic DNA, PB-52 strain was grown in GLM broth on a rotary shaker (150 rpm) at 28°C, pH 7.4 for 4 days. The cells were harvested by centrifugation (8000 rpm, 5 min), washed two times with sterile water and suspended in 800 μL lysis buffer (100 mM Tris-HCl, 20 mM EDTA, 250 mM NaCl, 2% SDS and 1 mg/mL lysozyme). 2 μL RNase was added and the sample was incubated at 37°C for 3 h. 2 μL proteinase K was added to the sample and incubated at 65°C for 30 min. 800 μL phenol, chloroform (1:1) was added and centrifuged (12,000 rpm, 5 min). The upper aqueous layer was collected, mixed with equal volume of chloroform, isoamyl alcohol (24:1) and centrifuged (12,000 rpm, 5 min). Again, the upper layer was collected, mixed with 0.1 volume sodium acetate along with 2 volume 96% ethanol and incubated at −20°C for 1 h and centrifuged (12,000 rpm, 15 min). The pellet was washed first with 300 μl 70% ethanol and then with 90% ethanol (8000 rpm, 10 min each). The pellet was air dried and suspended in 30 μL TE buffer (pH 7.7). DNA was analyzed by 1% agarose gel electrophoresis.

#### PCR amplification

16S rRNA gene was amplified using universal eubacterial primer set, 27F (5′-AGA GTT TGA TCC TGG CTC AG-3′), and 1492R (5′-GGT TAC CTT GTT ACG ACT T-3′) (Weisburg et al., 1991). PCR reactions were performed in Proflex PCR System (Applied Biosystems, USA) in a total volume of 50 μl reaction mixture containing 50 ng template DNA, 1X Taq DNA polymerase buffer, 1.5 mM MgCl_2_, 0.2 mM of each dNTP, 1 U Taq DNA polymerase enzyme and 0.2 μM of each primer. The thermal cycling conditions were programmed as follows: initial denaturation at 94°C for 5 min; followed by 35 cycles at 94°C for 30 s, 52°C for 30 s, 72°C for 1 min and final extension at 72°C for 10 min. The amplified products were determined by 1.8% (w/v) agarose gel electrophoresis. The amplified products were further purified using GenElute PCR Clean-Up Kit (Sigma Aldrich, USA) and the purified PCR products were sequenced by automated DNA sequencer with specific primers using the facility at Xcelris Genomics Lab Ltd. (Ahmedabad, India).

#### Phylogenetic analysis

Identification of phylogenetic neighbors and calculation of pairwise sequence similarities of 16S rRNA gene of PB-52 strain were carried out using BLASTN (Altschul et al., 1990) and EzTaxon server (http://www.eztaxon.org/; Kim et al., 2012). Top 14 reference sequences with highest scores were selected for multiple sequence alignment which was performed by CLUSTAL W program (Thompson et al., 1997). The phylogenetic tree was constructed by neighbor-joining method (Saitou and Nei, 1987) using MEGA version 4.0 (Tamura et al., 2007). The support of each clade was determined by bootstrap analysis performed with 1000 replications (Felsenstein, 1985). The GenBank accession number for the partial 16S rRNA gene sequence of PB-52 strain is KM406386.

### Evaluation of antimicrobial activity of PB-52 strain

#### Test microorganisms

The following test microorganisms were used for the experiment. Gram-positive bacteria: *S. aureus* MTCC 96, *S. aureus* MTCC 3160, MRSA ATCC 43300, *S. epidermidis* MTCC 435, *Bacillus subtilis* MTCC 441, *B. cereus* MTCC 1272, *B. megaterium* MTCC 8075, *Micrococcus luteus* MTCC 1538; Gram-negative bacteria: *Escherichia coli* MTCC 40, *E. coli* MTCC 739, *Serretia marcescens* MTCC 97, *Klebsiella pneumoniae* MTCC 3384, *K. pneumoniae* ATCC 13883, *Pseudomonas aeruginosa* MTCC 741, *P. aeruginosa* MTCC 424, *P. aeruginosa* MTCC 2582, *Proteus vulgaris* MTCC 426; Yeast: *C. albicans* MTCC 227, *C. tropicalis* MTCC 2208 and *C. albicans* ATCC 10231. All the MTCC strains were purchased from Microbial Type Culture Collection, CSIR-Institute of Microbial Technology, Chandigarh, India and ATCC cultures were purchased from HiMedia, Mumbai. The bacterial strains were cultured in nutrient agar medium at 37°C and yeasts strains were cultured on Sabouraud dextrose medium at 25°C. The test organisms were preserved at −70°C in glycerol stock vials for further study.

#### Antimicrobial activity assessment and media optimization

Strain PB-52 was screened for *in vitro* antimicrobial activity against test microorganisms by conventional spot inoculation method (Shomurat et al., 1979) on GLM agar medium after 8 days of incubation at 28°C. The inhibition zone was observed after 24–48 h incubation at 37°C for bacteria and at 25°C for yeasts. Each experiment was conducted in three replicates and the average size of PB-52 colony diameter and mean value of inhibition zone diameter (mm ± SD) of test microorganisms was calculated.

The selection of best culture medium for growth and antimicrobial activity of PB-52 was performed by growing the strain in different growth media such as GLM broth, Thronton's broth (K_2_HPO_4_, 1 g; KNO_3_, 0.5 g; MgSO_4_.2H_2_O, 0.2 g; CaCl_2_.H_2_O, 0.1 g; NaCl, 0.1 g; FeCl_3_, 0.01 g; aspargine, 0.5 g; distilled water, 1000 mL), CSPY-ME broth, Actinomycetes broth and nutrient broth. The strain PB-52 was grown with continuous shaking on a rotary shaker (150 rpm) at 28°C, pH 7.4 for 8 days. Antimicrobial potential was evaluated against *S. aureus* MTCC 96 by disc diffusion method (Bauer et al., 1966) since this test organism was found to be more sensitive during antimicrobial evaluation by spot inoculation method. The experiment was repeated three times and its mean value was recorded.

Crude extract of PB-52 strain was recovered from the culture filtrate by solvent extraction using ethyl acetate in 1:1 ratio (v/v). The ethyl acetate extract of PB-52 (EA-PB-52) obtained by evaporation of ethyl acetate was prepared by dissolving it in 10% dimethyl sulphoxide (DMSO) at a concentration of 1 mg/mL prior to antimicrobial bioassay. 20 μL EA-PB-52 was loaded on to sterile discs (6 mm diameter) placed on nutrient agar plates spread with bacterial test organisms (0.5 McFarland turbidity standards). Similarly, the experiment was conducted with *Candida* species on sabouraud dextrose agar plates. 10% DMSO loaded disc was used as a negative control while rifampicin (20 μg/disc) for bacteria and amphotericin B (30 μg/disc) for *Candida* species served as positive controls. Antimicrobial activity was observed after 24–48 h incubation at 37°C for bacteria and at 25°C for yeasts. Each experiment was conducted in three replicates and the mean value of inhibition zone diameter was calculated. Among all the different liquid media evaluated for antimicrobial activity, GLM was found to be the best medium for PB-52 strain; hence, it was selected as the production medium for further studies.

### Effect of temperature, pH and incubation period on growth and antimicrobial activity

GLM medium was inoculated with PB-52 strain to study the growth response and antimicrobial activity at different range of temperatures from 20 to 40°C at 150 rpm for 8 days at pH 7.4. To study the effect of pH, different ranges of acidic to alkaline pH-values (5–10) were used for the growth and antibiotic production in GLM broth at 28°C for 8 days. Similarly, incubation periods were observed up to 12 days to determine the optimum growth and antibiotic production of PB-52 in GLM broth at 28°C, pH 7.4. The antimicrobial activity of PB-52 was assessed against *S. aureus* MTCC 96 by disc diffusion method (El-Gendy et al., 2008; Thakur et al., 2009) in terms of diameter of inhibition zone.

### PCR amplification and sequencing of biosynthetic genes of PB-52 strain (PKS-I and NRPS)

Degenerate primers K1F (5′-TSA AGT CSA ACA TCG GBC A-3′) and M6R (5′- CGC AGG TTS CSG TAC CAG TA-3′) were used for amplification of PKS-I ketosynthase and methyl-malonyltransferase domain sequences and primers A3F (5′-GCS TAC SYS ATS TAC ACS TCS GG-3′) and A7R (5′-SAS GTC VCC SGT SCG GTA S-3′) were used for amplifications specific for NRPS adenylation domain sequences (Ayuso-Sacido and Genilloud, 2005). PCR reactions were performed in Proflex PCR System (Applied Biosystems, USA) in a final volume of 50 μl consisting of template DNA (50 ng), 1X Taq DNA polymerase buffer, MgCl_2_(1.5 mM), each dNTP (0.2 mM), 1 U Taq DNA polymerase enzyme and each primer (0.2 μM). For increasing the specificity, touchdown PCR was performed for the amplification of PKS-I genes. In touchdown PCR, annealing temperature was set 10°C above the expected annealing temperature (56.8°C) and decreased by 1°C every second cycle until a touchdown of 46.8°C, at which temperature 25 additional cycles were carried out. Denaturation was carried out at 94°C for 1 min, primer annealing was performed at the appropriate temperature (46.8°C) for 1 min, and primer extension at 72°C for 2 min followed by the final extension at 72°C for 10 min. The thermal cycling conditions for the amplification of NRPS genes were programmed as: initial denaturation at 94°C for 5 min; followed by 35 cycles at 94°C for 1 min, 63°C for 1 min, 72°C for 2 min, and final extension at 72°C for 10 min.

The amplified products were purified by GenElute PCR Clean-Up Kit (Sigma Aldrich, USA). The purified PCR products were sequenced by automated DNA sequencer with the specific PKS-I and NRPS primers using the sequencing facility at First BASE Laboratories, Malaysia. The resultant sequence was analyzed using BLAST N. The PKS-I (1040 bp fragment) and NRPS (647 bp fragment) gene sequences were deposited to GenBank under the accession number KU721843 and KU721842, respectively.

### Determination of minimum inhibitory concentration (MIC) of EA-PB-52

MIC was determined as illustrated by the Clinical Laboratory Standards Institute (CLSI; 2012) and Andrews (2001) with slight modifications using broth dilution method. An inoculum of 1 × 10^5^ CFU/mL of test microorganisms (log phase culture) was added to 5 mL of Mueller Hinton broth (for bacterial test organisms) and Sabouraud dextrose broth (for yeasts) in different test tubes and incubated at room temperature for 24–48 h. EA-PB-52 was dissolved in 10% DMSO (1000 μg/mL) and two-fold serial dilutions of the extract was prepared for MIC tests (62.5–0.975 μg/mL). MIC was determined after 48 h of incubation of the test microorganisms in the presence of EA-PB-52. 10 μl of the test microorganisms were removed from each tube and spread on Mueller Hinton agar plates/sabouraud dextrose agar plates. The growth of test organisms was observed after 24 h of incubation at 37°C for bacteria and 25°C for yeasts. MIC was recorded as the lowest concentration of the antimicrobial compound which inhibits the visible growth of the inoculated test microorganisms completely after 24–48 h. Control was prepared using 10% DMSO without antimicrobial agent which should be turbid (negative control) while control with standard antibiotic such as rifampicin, streptomycin, and amphotericin B should be clear (positive control).

### Rate of kill assay of EA-PB-52

Antimicrobial assay for the rate of killing of test microorganisms by EA-PB-52 was done in accordance with the description of Eliopoulos and Moellering (1996) using modified plating technique. EA-PB-52 was inoculated into 10 mL Mueller Hinton broth for bacterial test microorganisms and Sabouraud dextrose broth for yeasts at ½ × MIC, 1 × MIC and 2 × MIC. Broth with EA-PB-52 at the test concentrations without the test microorganisms and broth without EA-PB-52 with test organisms served as controls. 1 × 10^5^ CFU/mL inoculum was used to inoculate 10 mL of both control and test bottles. The bottles were incubated at 37°C for bacteria and 25°C for yeasts on an orbital shaker at 150 rpm. 100 μl aliquot was removed from the culture medium after 0, 4, and 8 h incubation to determine CFU/mL by the plate count technique (Cruickshank et al., 1975) by plating 25 μl of each of the dilutions. After incubation, emergent colonies were counted, CFU/mL was determined and compared with the count of the control culture without EA-PB-52. 10% DMSO was used as control. The experiments were conducted in replicates and the mean value was obtained. The results were expressed as positive or negative log_10_ values (Baltch et al., 1998).

### Interaction of EA-PB-52 with test microorganisms by SEM analysis

EA-PB-52 with strong antimicrobial activity was further studied for its possible effects on targeted cells of *P. aeruginosa* MTCC 741 and *C. albicans* MTCC 227 by SEM according to Supaphon et al. (2013) with slight modifications. Test microorganisms were grown overnight and then treated with 1 × MIC EA-PB-52. Cells were harvested after 24 h of incubation and washed with phosphate buffered saline (PBS), pH 7.4. The cells were fixed with 2.5% (v/v) glutaraldehyde in PBS for 2 h and dehydrated in series of increasing concentrations of acetone (30–100%, v/v) for 10 min. The cells were dried for 30–45 min and mounted onto steel stub with double-sided carbon tape. Samples were coated with a film of gold-palladium alloy under vacuum and scanned under scanning electron microscope (Zeiss Sigma VP, Germany).

### GC-MS analysis of EA-PB-52

Identification of the chemical compounds present in EA-PB-52 was analyzed using GC-MS as previously described (Ser et al., 2015b; Sun et al., 2015) with slight modifications. The sample was dissolved in spectroscopy-grade methanol and filtered through 0.2 μm filter. GC-MS analysis was performed on Shimadzu GC 2010 plus with triple quadrupole MS (TP-8030) fitted with EB-5 MS column (length- 30 m, thickness–0.25 μm, internal diameter–25 mm). The oven programme started at 40°C, held for 5 min and then ramped at 10°C/min to 280°C, held for 10 min and then again raised to 285°C at 5°C/min and finally held for 10 min. Sample was injected at 300°C using helium as carrier gas (1 mL/min), split at the ratio of 1:20. The mass spectrometer was operated in the electron ionization (EI) mode at 70 eV with a continuous scan from 45 to 600 m/z. The peaks were identified by matching the mass spectra with the National Institute of Standards and Technology (NIST, USA) library.

### Detection of polyenic or non-polyenic antimicrobial activity of EA-PB-52

The polyene like activity of EA-PB-52 was determined by ergosterol agar plate method by using ergosterol as the reversal agent to test for its ability to reverse the inhibition of *C. albicans* MTCC 227 caused by the metabolite (Thakur et al., 2007). EA-PB-52 was dissolved in methanol and its absorption spectrum was scanned in the UV-vis region (200–800 nm) by using a Nanodrop 2000c UV-vis spectrophotometer (Thermo Fisher Scientific, USA).

### Statistical analysis

All experiments were performed in biological triplicate and repeated for three times. The data was expressed as the mean ± standard deviation of mean of the three replicates. Duncan's multiple range test was performed to compare that the sample means differ significantly from each other at a significant level of *P* < 0.05 (Gomez and Gomez, 1984).

## Results

### Characterization of PB-52 strain

**Figure 1:**
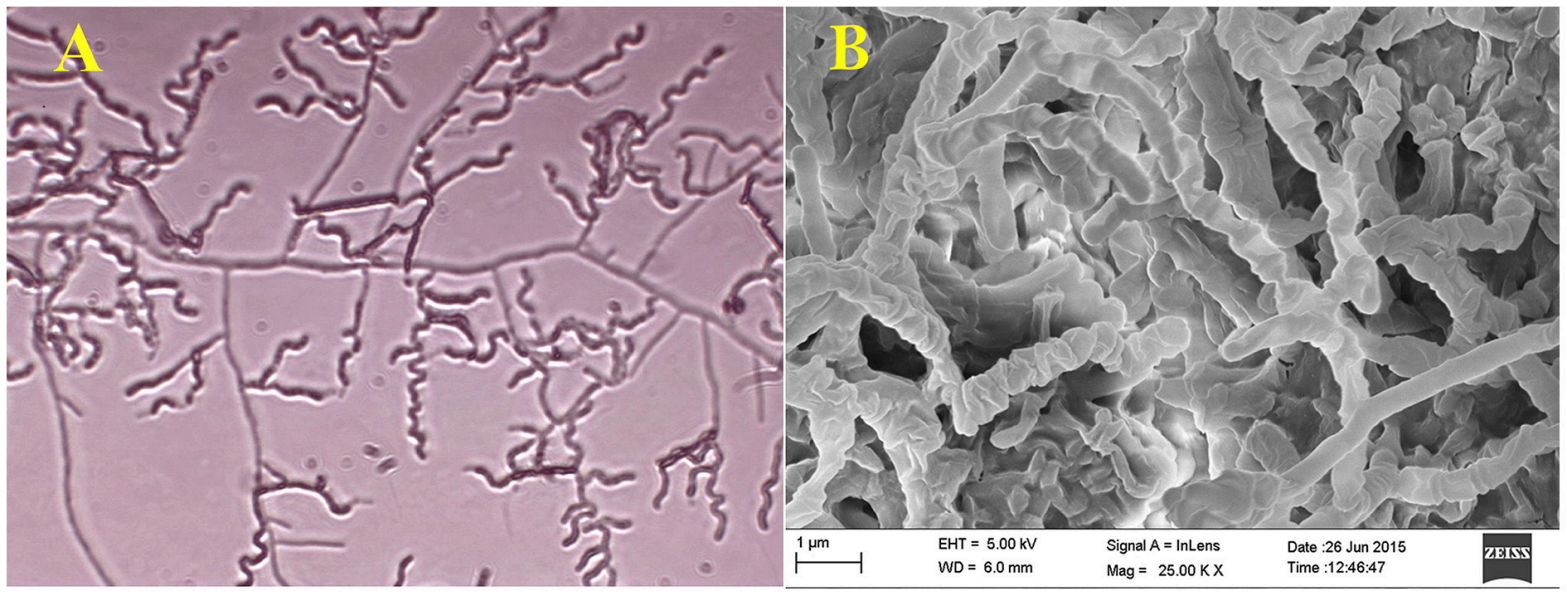
**Microscopic view showing spore chain of ***Nocardia*** sp. PB-52 on GLM agar (A) Micro morphology using cover slip insertion method; (B) Scanning electron micrograph view**.

Actinomycete strain PB-52 isolated from soil samples of Pobitora Wildlife Sanctuary of Assam, India, was aerobic, Gram-positive, and filamentous in nature (Figure 1A). The branched vegetative hyphae were light brown in color while aerial mycelium with orange color was sparse with a patchy distribution. The strain produced faint brown soluble pigment after incubation for more than 20 days at 28°C. SEM analysis revealed that the aerial mycelia formed long, straight to rectiflexibiles spore chains with rugose spore surface (Figure 1B). The cultural characteristics of PB-52 strain in different media are shown in Table 1, where the strain showed good growth on all the media except Mueller Hinton agar and Omeliansky's agar medium. The strain was found to grow between the temperature range of 20–42°C, pH 5–11 with optimal growth temperature at 28°C, pH 7.4, and in the presence of upto 5% NaCl (w/v) with optimum at 1–3% NaCl. The morphological, physiological, and biochemical characteristics of PB-52 and its antibiotic sensitivity are shown in Table 2. The whole-cell hydrolysates of PB-52 were rich in the meso-diaminopimelic acid along with galactose and arabinose as characteristic whole cell sugars.

**Table 1:** **Cultural characteristics of ***Nocardia*** sp. PB-52 on different media**.

**Medium**	**Aerial mycelium color**	**Substrate mycelium color**	**Diffusible pigment**	**Growth**
Actinomycetes isolation agar	Brown	Dark brown	−	+++
Streptomyces agar	Light orange	Orange	−	++
GLM medium	Orange	Light brown	Faint Brown	+++
CSPY-ME medium	Pink	Brown	−	+++
Mueller Hinton Agar	Light brown	Brown	−	+
Nutrient agar	White	Cream	−	++
Sabouraud Dextrose Agar	Orange	Cream	−	++
Thronton's medium	Orange	Brown	−	++
Omeliansky's agar	White	Brown	−	+
ISP 2	Pink	Orange	−	+++
ISP 3	White	Brown	−	++
ISP 4	White	Cream	−	+++
ISP 7	Orange	Cream	−	++

*+++, Good growth; ++, Moderate growth; +, Poor growth; −, No growth*.

**Table 2:** **Morphological, physiological, and biochemical characteristics of ***Nocardia*** sp. PB-52**.

**Characteristics**	**Result**
**MORPHOLOGICAL**	
Aerial mycelium color	Orange
Substrate mycelium color	Brown
Diffusible pigment	Faint brown
Melanin pigment	–
Spore chain morphology	Straight to rectiflexibiles
Spore surface	Rugose
**PHYSIOLOGICAL**	
Temperature range for growth	20–42°C
Optimum temperature for growth	28°C
pH range for growth	5–11
Optimum pH for growth	7.4
NaCl tolerance	Up to 5%
**BIOCHEMICAL**	
Gram reaction	Positive
Utilization of	
Glucose	+
Fructose	+
Arabinose	−
Mannitol	+
Inositol	+
Adonitol	+
Galactose	−
Sucrose	+
Xylose	+
Lactose	+
Maltose	+
Starch	+
Glycerol	+
Erythritol	−
Sorbitol	−
Rhamnose	−
Gluconate	−
Carboxy methyl cellulose	+
Citrate	−
Urea	+
Ammonium chloride	+
Ammonium sulfhate	+
Sodium nitrate	+
Glycine	+
Asparagine	+
Casein	−
Tween 20	−
Tween 80	+
Nitrate reduction	+
Gelatin liquefaction	−
Cell wall amino acids	Meso-diaminopimelic acid
Cell wall sugars	Arabinose, galactose
**ANTIBIOTIC SENSITIVITY (**μ**g/disc)**	
Vancomycin (30)	S
Chloramphenicol (30)	S
Oxacillin (1)	R
Ciprofloxacin (5)	S
Co-trimoxazole (25)	R
Streptomycin (10)	S
Methicillin (5)	S
Ampicillin (10)	R
Penicillin-G (10)	S
Gentamicin (120)	R
Nalidixic acid (30)	S
Erythromycin (5)	S
Norfloxacin (10)	S
Amphotericin B (100)	R
Clotrimazole (10)	S
Fluconazole (25)	R
Itraconazole (10)	R
Ketoconazole (10)	S
Nystatin (100)	R

*+, Positive for test; −, Negative for test; S for Sensitivity; R for Resistant*.

**Figure 2:**
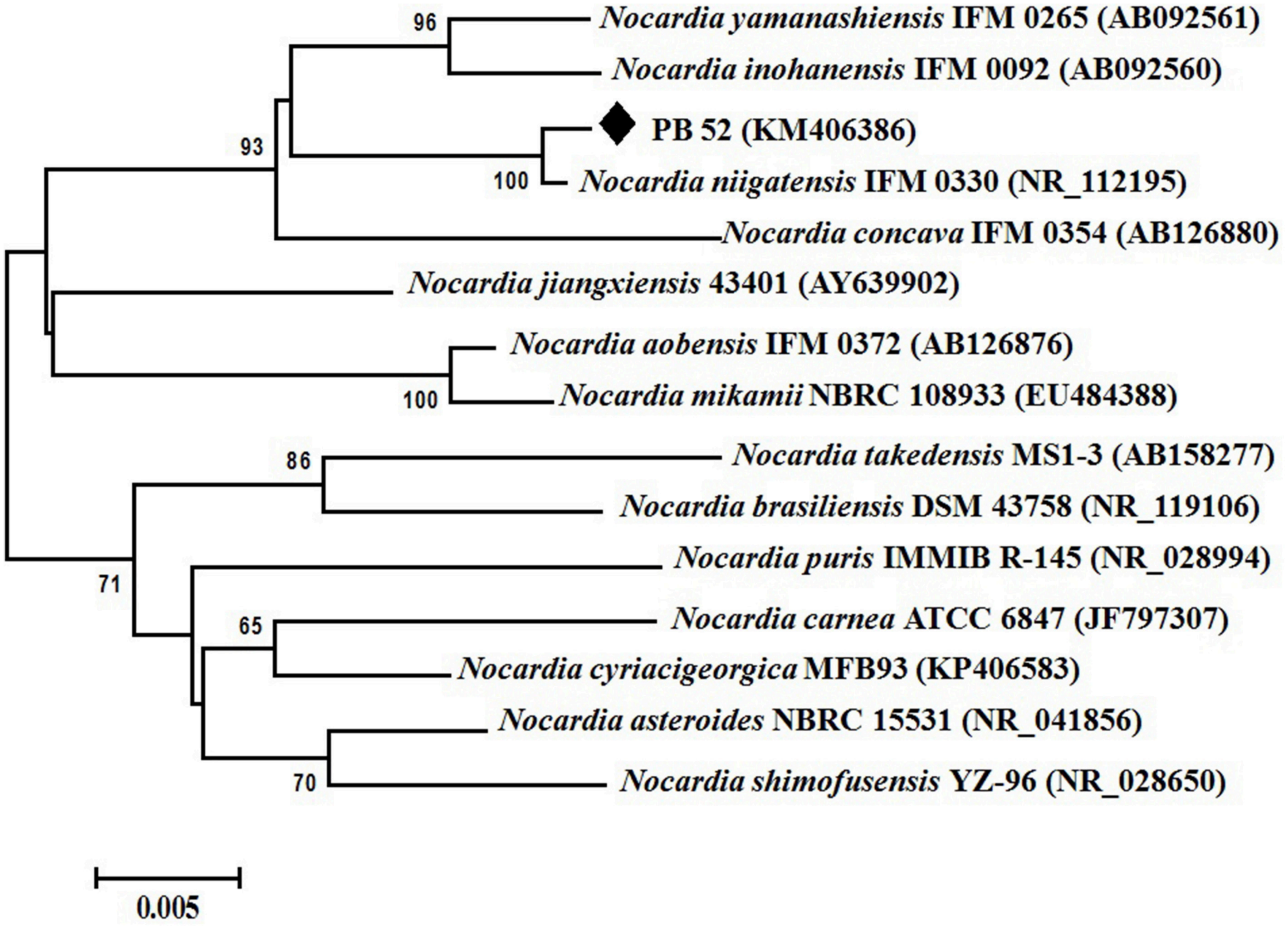
**Phylogenetic tree of ***Nocardia*** sp. PB-52 and the closest ***Nocardia*** species showing phylogenetic relationships based on the 16S rRNA gene sequences using neighbor-joining method**. Bootstrap percentages based on 1000 resamplings are listed at nodes, only values above 50% are given. Bar, 0.005 substitutions per nucleotide position.

Partial 16S rRNA gene sequence (1259 nucleotides) of PB-52 strain was determined and submitted to NCBI GenBank database under the accession number KM406386. The strain showed highest 16S rRNA gene sequence similarities with *Nocardia niigatensis* IFM 0330 (NR_112195; 99.7%). The phylogenetic tree also indicated its closest similarity to *N. niigatensis* based on Neighbor-joining method (Figure 2). The phenotypic and genomic data indicated that PB-52 strain represented a strain of the genus *Nocardia* for which the strain was referred to as *Nocardia* sp. strain PB-52.

### Antimicrobial potential of PB-52 strain

During the screening of PB-52 strain for drug discovery by spot inoculation method in GLM agar medium, it was observed that PB-52 exhibited excellent activity by inhibiting Gram-positive bacteria, Gram-negative bacteria and yeasts. The average size of the colony of PB-52 in GLM medium was (7 ± 2) mm in diameter after 8 days of incubation at 28°C. The maximum mean value of inhibition zones (mm ± SD) was found against *S. aureus* MTCC 96 (inhibition zone diameter of 36 ± 0.8), followed by *E. coli* MTCC 40 (31 ± 1.2) and MRSA ATCC 43300 (30 ± 0.8). Maximum growth inhibition among yeast test pathogens was observed in *C. albicans* MTCC 227 (27 ± 0.4). The detailed antimicrobial profiles in GLM agar medium are shown in Table 3.

**Table 3:** **Antimicrobial activity and MIC (μg/mL) of ***Nocardia*** sp. PB-52 by broth dilution method**.

**Test microorganisms**	^*^Zone of inhibition (mm)	**MIC of EA-PB-52 (μg/mL)**	**MIC of Rif (μg/mL)**	**MIC of Strep (μg/mL)**	**MIC of Amp B (μg/mL)**
**GRAM-POSITIVE BACTERIA**					
*S. aureus* MTCC 96	36^k^±0.8	>0.975	>1.95	>6.25	NA
*S. aureus* MTCC 3160	29^hij^±0.8	>1.95	>1.95	>12.5	NA
*S. epidermidis* MTCC 435	23^de^±1.6	>7.81	>3.125	>6.25	NA
*B. subtilis* MTCC 441	28^ghi^±0.8	>1.95	>1.95	>6.25	NA
*B. cereus* MTCC 1272	22^d^±0.4	>7.81	>6.25	>12.5	NA
*B. megaterium* MTCC 8075	26^fg^±0.4	>3.9	>3.12	>3.12	NA
*M. luteus* MTCC 1538	22^d^±1.6	>7.81	>0.97	>6.25	NA
MRSA ATCC 43300	30^ij^±0.8	>1.95	>25	>50	NA
**GRAM-NEGATIVE BACTERIA**					
*E. coli* MTCC 40	31^j^±1.2	>3.9	>6.25	>3.12	NA
*E. coli* MTCC 739	25^ef^±0.4	>3.9	>50	−	NA
*S. marcescens* MTCC 97	18^b^±1.2	>31.2	>12.5	>3.12	NA
*K. pneumoniae* MTCC 3384	27^fgh^±1.6	>1.95	>25	−	NA
*K. pneumoniae* ATCC 13883	15^a^±1.6	>62.5	>50	−	NA
*P. aeruginosa* MTCC 741	29^hij^±0.8	>1.95	>25	>25	NA
*P. aeruginosa* MTCC 424	19^bc^±1.2	>15.6	−	>12.5	NA
*P. aeruginosa* MTCC 2582	26^fg^±0.8	>3.9	>50	>25	NA
*P. vulgaris* MTCC 426	17^a^±0.8	>31.25	>25	>6.25	NA
**YEAST**					
*C. albicans* MTCC 227	27^fgh^±0.4	>1.95	NA	NA	>0.97
*C. tropicalis* MTCC 2208	23^de^±0.2	>7.81	NA	NA	>0.48
*C. albicans* ATCC 10231	21^cd^±1.6	>7.81	NA	NA	>1.95

* *Zone of inhibition by spot inoculation method on GLM agar medium. Average size of colony of PB-52 in GLM agar was (7 ± 2) mm in diameter after 8 days of incubation at 28°C*.

*Zone of inhibition values are given as mean ± SD (n = 3). Values having different superscripts (a-k) differ significantly (P < 0.05)*.

*Rif, Rifampicin (antibacterial agent); Strep, Streptomycin (antibacterial agent); Amp B, Amphotericin B (antifungal agent); NA, not applicable; −, No activity*.

**Figure 3:**
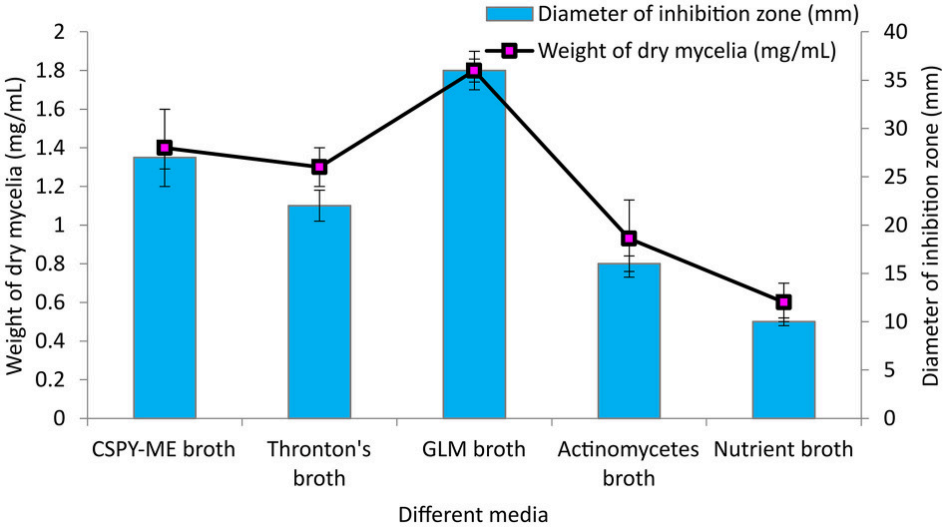
**Effect of different culture media on growth and antimicrobial activity assessed in terms of diameter of inhibition zone by ***Nocardia*** sp. PB-52 (Test organism: ***S. aureus*** MTCC 96)**.

**Figure 4:**
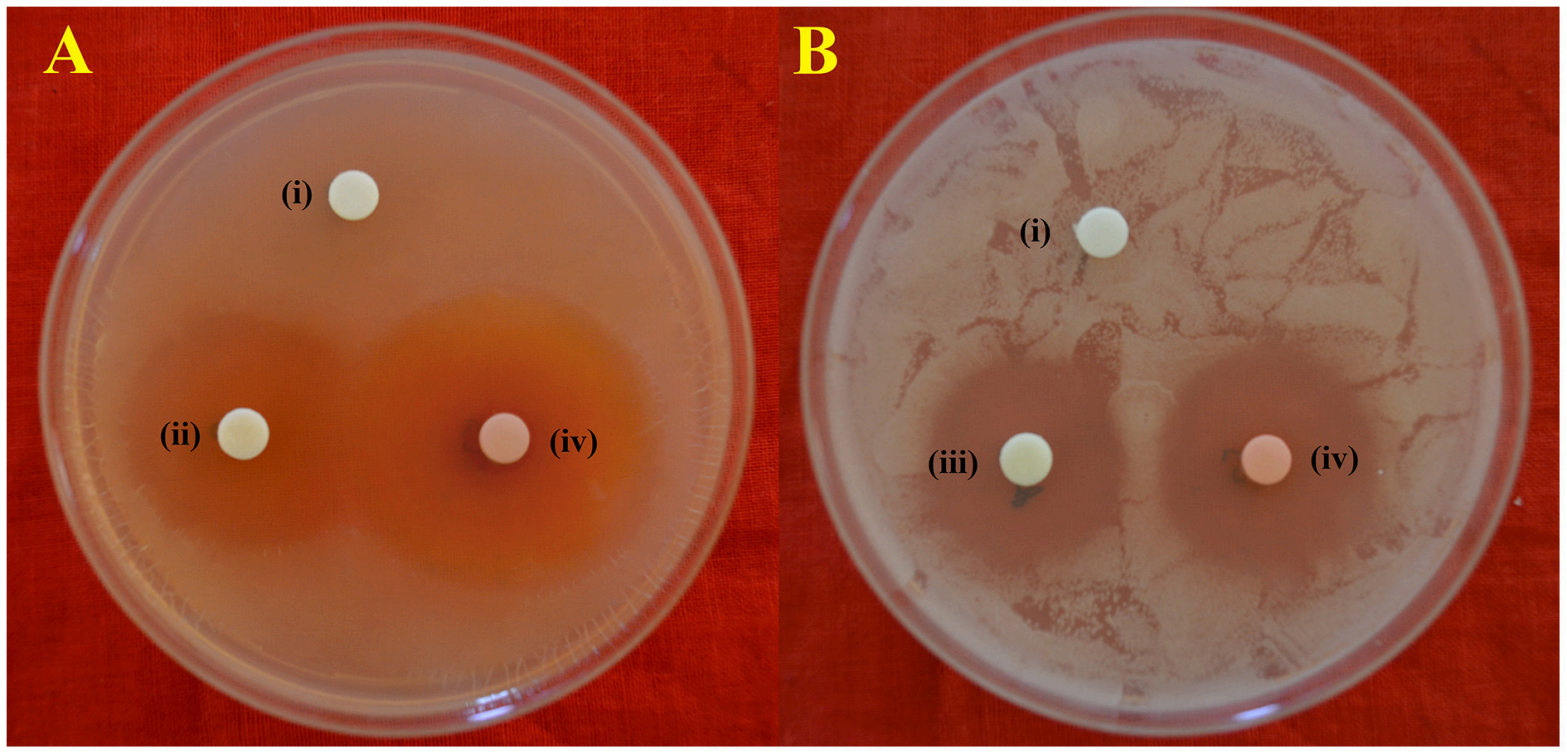
*****In-vitro*** antimicrobial activity of EA-PB-52 following disc diffusion method against (A) ***S. aureus*** MTCC 96 (B) ***C. albicans*** MTCC 227 (i) 10% DMSO (negative control), (ii) 20 μg/disc rifampicin (positive control), (iii) 30 μg/disc amphotericin B (positive control), (iv) 20 μg/disc EA-PB-52**.

Among the different liquid culture media evaluated for the production of the bioactive compounds in shake flask condition, GLM was found to be most appropriate for growth and antimicrobial activity assessed in terms of diameter of inhibition zone by PB-52 strain (Figure 3). The culture filtrate of PB-52 grown on GLM medium exhibited maximum zone of inhibition against *S. aureus* MTCC 96 followed by the culture filtrate grown on CSPY-ME broth while lowest activity was observed with the one grown on nutrient broth. The antimicrobial activity of EA-PB-52 along with the controls (10% DMSO as negative control and standard antibiotics as positive control) against *S. aureus* MTCC 96 and *C. albicans* MTCC 227 is shown in Figure 4.

### Effect of cultural parameters on growth and antimicrobial activity

**Figure 5:**
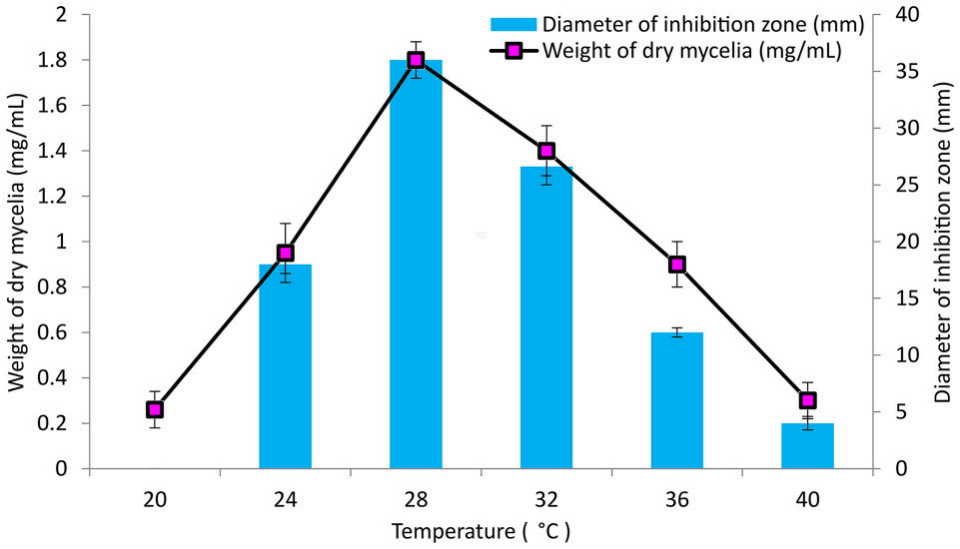
**Effect of temperature on growth and antimicrobial activity assessed in terms of diameter of inhibition zone by ***Nocardia*** sp. PB-52 (Test organism: ***S. aureus*** MTCC 96)**.

**Figure 6:**
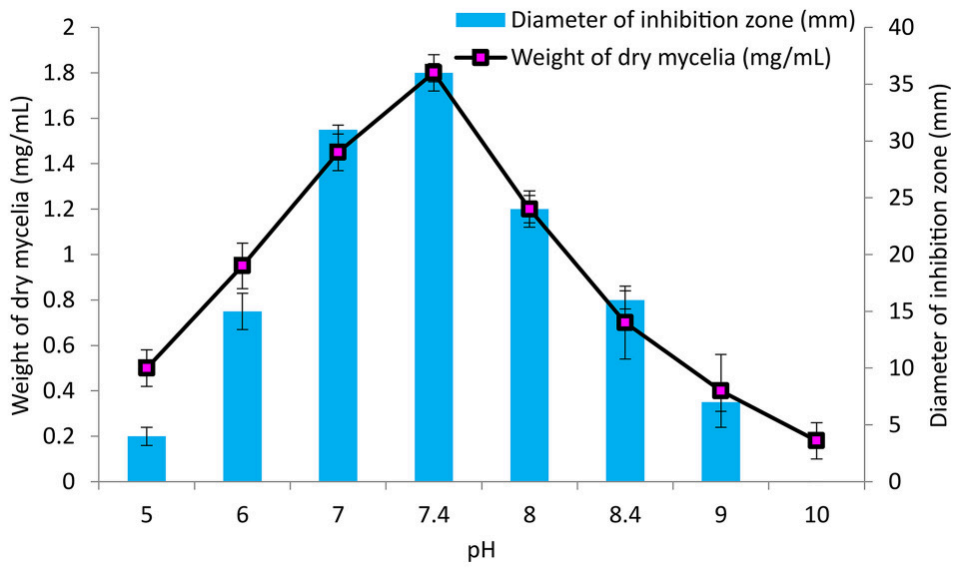
**Effect of pH on growth and antimicrobial activity assessed in terms of diameter of inhibition zone by ***Nocardia*** sp. PB-52 (Test organism: ***S. aureus*** MTCC 96)**.

**Figure 7:**
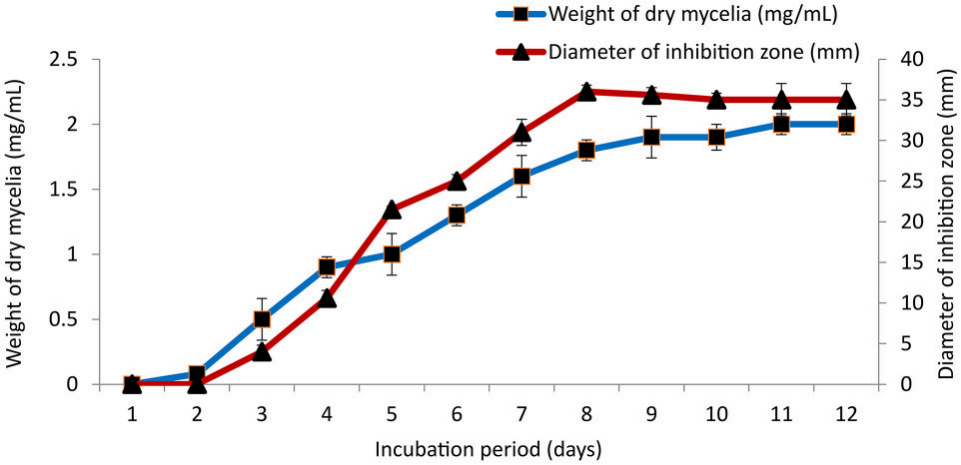
**Effect of incubation period on growth and antimicrobial activity assessed in terms of diameter of inhibition zone by ***Nocardia*** sp. PB-52 (Test organism: ***S. aureus*** MTCC 96)**.

The culture conditions for growth and antimicrobial evaluation by PB-52 strain was studied on GLM medium. PB-52 displayed a narrow range of incubation temperature for its good growth and production of antibiotics. 28°C was found to be the optimum temperature for highest growth and maximum antimicrobial activity by the strain (Figure 5). The organism appeared to be mesophilic in terms of its growth at optimum temperature. However, poor growth and decreased antibiotic production were evident at higher incubation temperature. The maximum growth as well as highest antibiotic production was obtained at pH-value of 7.4 (Figure 6). However, adverse growth and antibiotic production were observed at pH-values above and below neutral. Maximum incubation period of 8 days is required for best growth and antibiotic production under shake flask conditions (Figure 7). Antimicrobial activity by PB-52 strain was assessed in terms of diameter of inhibition zone.

### Analysis of PKS-I and NRPS genes

**Figure 8:**
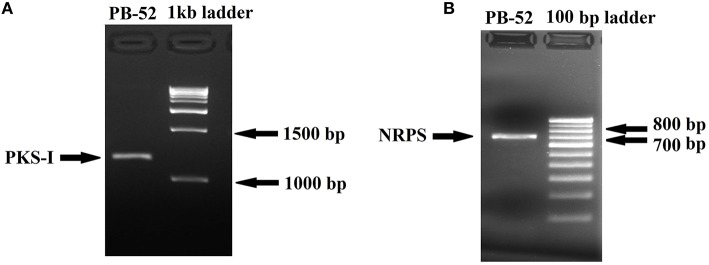
**Agarose gel electrophoresis of PCR amplified products of ***Nocardia*** sp. PB-52. (A) Selective amplification of PKS-I using K1F/M6R specific primers; (B) Selective amplification of NRPS using A3F/A7R specific primers**.

Polyketide synthase (PKS) and non-ribosomal peptide synthetase (NRPS) are biosynthetic enzymes responsible for the production of different classes of bioactive molecules in Actinomycetes (Hodges et al., 2012). PCR amplification of PKS-I gene of the strain PB-52 resulted in a band size of 1200–1400 bp fragments (Figure 8A) using K1F/M6R primers corresponding to PKS-I ketosynthase and methyl-malonyl-CoA transferase modules of the polyketide synthase gene. Sequencing of the PKS-I gene fragment yielded a sequence length of 1040 bp (NCBI accession no. KU721843). PB-52 strain showed highest PKS-I gene sequence similarities (89%) with *Streptomyces olivoviridis* strain O855 clone 22 modular polyketide synthase gene (NCBI accession no. FJ405974). The NRPS amplicon was found to be 700 bp size (Figure 8B) using A3F/A7R specific primers for NRPS genes targeting NRPS adenylation domain sequences. Sequencing of the NRPS gene fragment yielded 647 bp sequence length (NCBI accession no. KU721842). NRPS sequence of PB-52 showed highest similarities (76%) to *Streptomyces* sp. Sp080513GE-23 gene for non-ribosomal peptide synthetase (NCBI accession no. AB492018). The closest similarity of PKS-I and NRPS sequences of PB-52 to the closest relatives in GenBank is shown in Table S1.

### MIC of EA-PB-52

MIC values of EA-PB-52 ranging from 62.5 to 0.975 μg/mL were performed against all the test microorganisms by broth dilution method. The quantitative efficiency of EA-PB-52 against these organisms was estimated as MIC and it has got lowest MIC against *S. aureus* MTCC 96 (0.975 μg/mL) whereas highest was recorded against *K. pneumoniae* ATCC 13883 (62.5 μg/mL; Table 3). According to CLSI recommendations for MIC, *K. pneumoniae* ATCC 13883 was found to be resistant to EA-PB-52 (MIC: 62.5 μg/mL) since ≤ 8 μg/mL was taken as susceptible, ≤ 16 μg/mL as intermediate and ≥32 μg/mL as resistant (CLSI, 2012). It was observed that 10% DMSO (control) had no inhibitory effect on the test microorganisms.

### Time-kill assay

The time-kill assay was conducted for EA-PB-52 against all the test microorganisms. Table 4 shows the changes in the log_10_ CFU/mL of viable colonies of the test microorganisms following exposure to different concentration of EA-PB-52 which is expressed in terms of log_10_ CFU/mL change. It is based on the conventional bactericidal activity standard, i.e., 3 log_10_ CFU/mL or greater reduction in the viable colony count (Pankey and Sabath, 2004). After incubating the test microorganisms for 4 h with 1 × MIC and 2 × MIC of EA-PB-52, the average log reduction in the viable cell count ranged between −0.157 log_10_ and 1.897 log_10_ CFU/mL. While after 8 h of incubation, the average log reduction in the viable cell count ranged between −2.092 log_10_ and 0.864 log_10_ CFU/mL. After 4 h of incubation of the test microorganisms with 1 × MIC, the average log reduction in the viable cell count ranged between 0.947 log_10_ and 1.897 log_10_ while incubating the test microorganisms with 2 × MIC resulted in the average log reduction in the viable cell count ranging between −0.157 log_10_ and 0.891 log_10_. After 8 h of incubation with 1 × MIC resulted in average log reduction in the viable cell count between 0.342 log_10_ and 0.864 log_10_ while 2 × MIC resulted in the average log reduction in the viable cell count ranging between −2.092 log_10_ and −1.213 log_10_. The highest log reduction in cell density with EA-PB-52 was observed in *S. aureus* MTCC 96 (−2.092 log_10_ CFU/mL) followed by *E. coli* MTCC 40 (−1.917 log_10_ CFU/mL) and MRSA ATCC 43300 (−1.908 log_10_ CFU/mL), while least log reduction in cell density was observed in *K. pneumoniae* ATCC 13883 (−1.213 log_10_ CFU/mL) followed by *P. vulgaris* MTCC 426 (−1.325 log_10_ CFU/mL). No inhibitory effect was observed with 10% DMSO (control) on the test microorganisms.

**Table 4:** *****In vitro*** time-kill assessment of EA-PB-52 against the test microorganisms**.

**Test microorganisms**	**log**_**10**_ **kill (1/2)** × **MIC**			**log**_**10**_ **kill 1** × **MIC**			**log**_**10**_ **kill 2** × **MIC**		
	**0 h**	**4 h**	**8 h**	**0 h**	**4 h**	**8 h**	**0 h**	**4 h**	**8 h**
**GRAM-POSITIVE BACTERIA**									
*S. aureus* MTCC 96	2.452	2.772	4.330	2.456	0.947	0.342	2.468	–0.157	–2.092
*S. aureus* MTCC 3160	2.510	3.245	3.998	2.487	1.263	0.582	2.433	0.422	–1.775
*S. epidermidis* MTCC 435	2.853	3.421	4.227	2.610	1.512	0.539	2.717	0.531	–1.717
*B. subtilis* MTCC 441	2.329	3.022	4.172	2.311	1.235	0.492	2.352	0.544	–1.842
*B. cereus* MTCC 1272	2.115	2.914	4.013	2.302	1.429	0.483	2.196	0.436	–1.741
*B. megaterium* MTCC 8075	2.277	3.510	4.734	2.256	1.197	0.548	2.290	0.282	–1.869
*M. luteus* MTCC 1538	2.314	3.219	3.816	2.334	1.315	0.513	2.105	0.587	–1.512
MRSA ATCC 43300	2.412	2.868	3.797	2.249	1.206	0.429	2.270	0.124	–1.908
**GRAM-NEGATIVE BACTERIA**									
*E. coli* MTCC 40	2.212	2.842	3.714	2.368	1.191	0.404	2.292	–0.112	–1.917
*E. coli* MTCC 739	2.401	3.124	3.874	2.427	1.374	0.473	2.218	0.677	–1.880
*S. marcescens* MTCC 97	2.316	2.997	4.512	2.212	1.529	0.597	2.307	0.528	–1.563
*K. pneumoniae* MTCC 3384	2.312	3.046	4.147	2.299	1.434	0.501	2.333	0.648	–1.693
*K. pneumoniae* ATCC 13883	2.216	3.934	4.729	2.137	1.897	0.864	2.296	0.891	–1.213
*P. aeruginosa* MTCC 741	2.142	3.013	3.976	2.312	1.463	0.413	2.133	0.432	–1.436
*P. aeruginosa* MTCC 424	2.314	3.814	5.214	2.367	1.545	0.456	2.121	0.612	–1.517
*P. aeruginosa* MTCC 2582	2.202	2.889	4.318	2.311	1.385	0.567	2.273	0.418	–1.493
*P. vulgaris* MTCC 426	2.013	3.214	4.813	2.213	1.662	0.727	2.115	0.723	–1.325
**YEAST**									
*C. albicans* MTCC 227	2.104	3.116	3.937	2.204	1.229	0.435	2.316	0.499	–1.865
*C. tropicalis* MTCC 2208	2.049	3.313	4.712	2.014	1.575	0.541	2.179	0.583	–1.630
*C. albicans* ATCC 10231	2.098	3.236	4.213	2.212	1.305	0.512	2.513	0.513	–1.518

### SEM analysis

**Figure 9:**
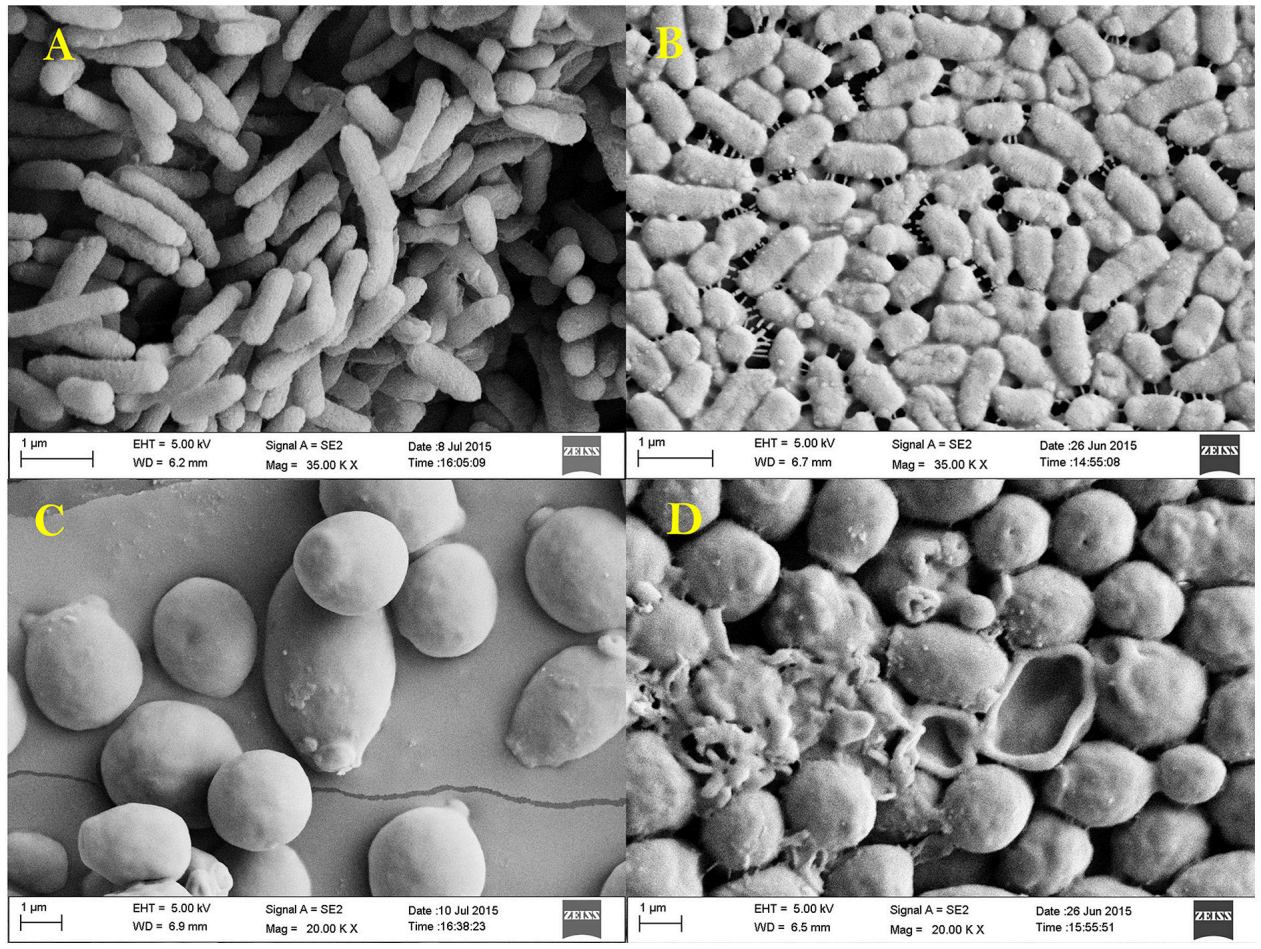
**Scanning electron micrograph showing the effect of 1 × MIC EA-PB-52 against ***P. aeruginosa*** MTCC 741 (A) without treatment, (B) treatment with EA-PB-52; and against ***C. albicans*** MTCC 227 (C) without treatment, (D) treatment with EA-PB-52**.

Antibacterial and anti-candidal activity of the EA-PB-52 was evaluated against *P. aeruginosa* MTCC 741 and *C. albicans* MTCC 227 by SEM analysis. SEM study revealed that treatment of the test microorganisms with 1 × MIC EA-PB-52 showed considerable morphological alterations in the cells including shrinkage and deformity leading to prominent cell shape loss and integrity. The control cells treated with 10% DMSO appeared smooth with intact cell surface (Figure 9).

### GC-MS analysis

**Figure 10:**
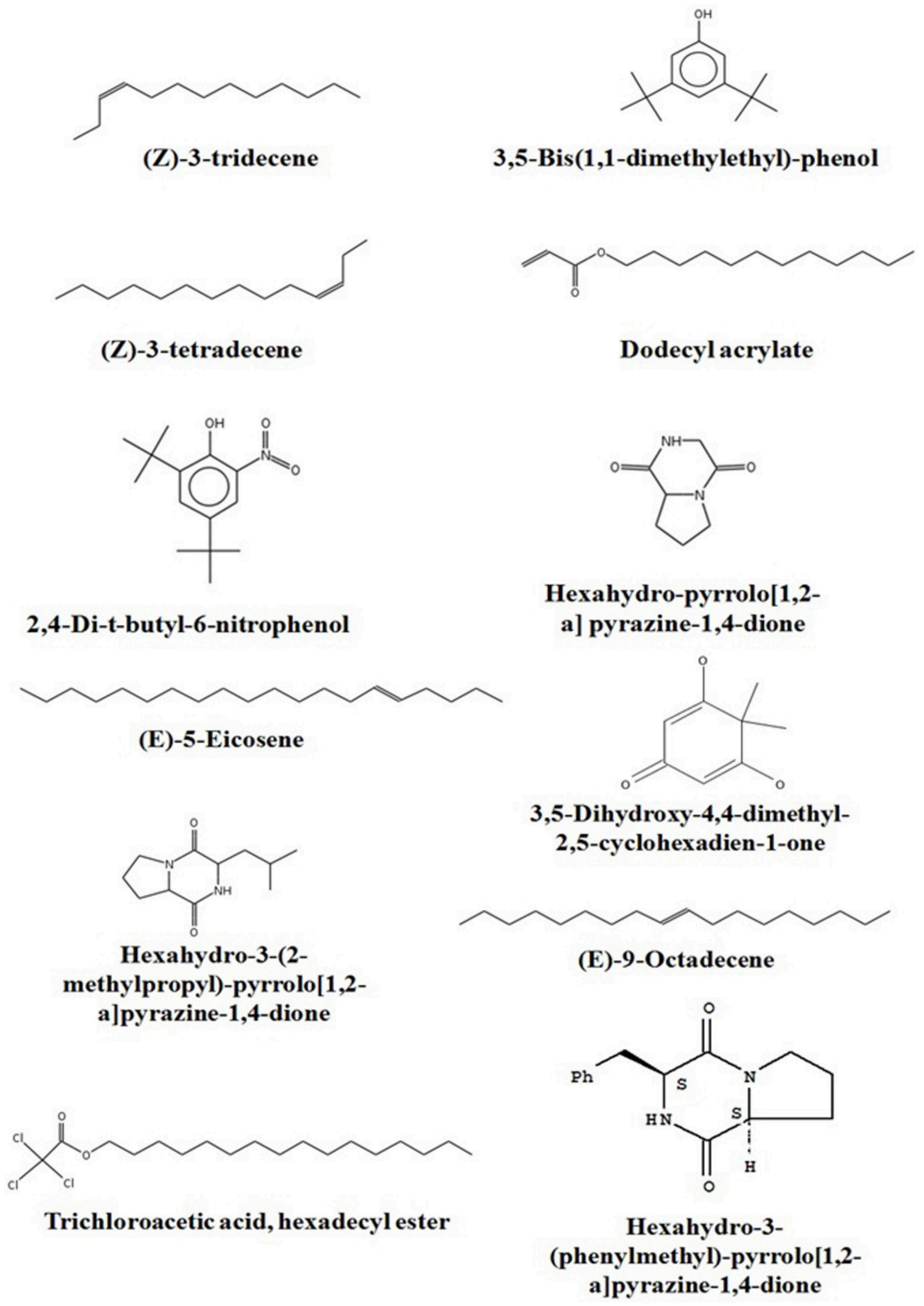
**Chemical structures of the identified compounds from EA-PB-52**.

Chemical composition of EA-PB-52 was done using GC-MS. Twelve chemical compounds were identified by comparison of their mass spectra with the NIST library based on their retention time, molecular weight, and molecular formula shown in Table 5 and the chemical structures were illustrated in Figure 10. The peak area of the compound is directly proportional to its quantity in EA-PB-52 (Figure S1).

**Table 5:** **Chemical compounds detected in EA-PB-52 by GC-MS analysis**.

**Compound name**	**Similarity (%)**	**RT**	**MW**	**Area (%)**	**Nature of compound**	**Activity**	**References**
(Z)-3-tridecene	95	18.27	182	5.14	Hydrocarbon	No activity reported	
3,5-bis (1,1-dimethylethyl)-phenol	91	19.93	206	34.43	Phenolic compound	No activity reported	
(Z)-3-tetradecene	95	20.94	196	7.90	Hydrocarbon	Antimicrobial	Natarajan and Dhas, 2014
Dodecyl acrylate	96	22.13	240	6.01	Ester	Antibacterial	Manilal et al., 2009
2,4-di-t-butyl-6-nitrophenol	63	22.22	251	4.32	Phenolic compound	Antimicrobial	Gutierrez et al., 2009
Hexahydro-pyrrolo[1,2-a]pyrazine-1,4-dione	90	23.11	154	5.03	Pyrrolizidine	Antimicrobial Antioxidant	Narasaiah et al., 2014; Ser et al., 2015a
(E)-5-Eicosene	95	23.32	280	5.07	Hydrocarbon	Antimicrobial	Elavarasi et al., 2014
3,5-dihydroxy-4,4-dimethyl-2,5-cyclohexadien-1-one	81	24.08	154	3.60	Quinone	No activity reported	
Hexahydro-3-(2-methylpropyl)-pyrrolo[1,2-a]pyrazine-1,4-dione	89	25.11, 25.17	210	9.04	Pyrrolizidine	Antimicrobial	Manimaran et al., 2015
(E)-9-Octadecene	94	25.47	252	2.21	Hydrocarbon	Antimicrobial	Cao et al., 2014; Okwu and Ighodaro, 2010
Trichloroacetic acid, hexadecyl ester	87	27.42	387	1.03	Acid	Cytotoxic, Antioxidant	Luo et al., 2014
Hexahydro-3-(phenylmethyl)-pyrrolo[1,2-a]pyrazine-1,4-dione	82	29.08	244	16.22	Pyrrolizidine	Antimicrobial, Nematicidal	Dashti et al., 2014; Wang et al., 2014

*RT is retention time; MW is molecular weight of compounds*.

### Detection of polyenic or non-polyenic antimicrobial activity of EA-PB-52

The ergosterol test of EA-PB-52 showing antimicrobial activity indicated the absence of polyene class of antibiotics. The nature of the metabolite was thus found to be non-polyene. The UV-vis spectrum of the EA-PB-52 in methanol showed the presence of three distinct peaks at 247, 260, and 296 nm where maximum absorption peak was observed at 296 nm (Figure S2).

## Discussion

Actinomycetes remain as the most proliferant producers of small molecule diversity including antimicrobials, diverse group of enzymes and useful secondary metabolites with an incredible variety of biological activities (Berdy, 2005). Depending on the abundance of actinomycetes in the soil, 99% of the diverse species has still been unexplored (Davies, 1999). Isolation of these novel species from unexplored habitats increases the possibility of discovery of new types of microbial products with new types of activities. Many ecosystems have still been poorly investigated and thus, there is a remarkable likelihood to exploit novel microorganisms with diverse biological activities from such ecosystems. There are several reports available regarding the study of metabolic pathways leading to the production of novel secondary metabolites from different microorganisms exploited from untapped ecosystems (Glover, 1995; Tiwari et al., 2014). Actinomycetes from diverse environments have been explored in the last few decades for their capability to produce a wide variety of natural bioactive compounds (Mitra et al., 2008). Isolation of actinomycetes for the production of antimicrobial secondary metabolites from the forest ecosystems of Northeast India is reported by several researchers (Thakur et al., 2007; Talukdar et al., 2012; Sharma et al., 2014; Passari et al., 2015). After literature review, it has been found that Pobitora Wildlife Sanctuary of Assam, India is an unexplored ecosystem and no considerable data could be found regarding bioactivity prospective of its microflora. Thus, we have an additional advantage by isolating a *Nocardia* strain selectively designated as PB-52 having broad spectrum bioactivity against a wide range of pathogens including MRSA from this unexplored habitat. Pobitora Wildlife Sanctuary is a part of the mega biodiversity hotspot which indicates that it is a very dynamic ecosystem where the occurrence of novel microflora may be very likely (Myers et al., 2000). This study signifies the first report on genus *Nocardia* isolated from this untouched habitat.

Based on the comparative study of 16S rRNA gene sequence and phylogenetic relationship, PB-52 strain lies in clade with *N. niigatensis* IFM 0330 (NR_112195). PB-52 shared 99.7% sequence similarity with type strain *N. niigatensis* IFM 0330 with 77 nucleotide differences, where PB-52 has a stretch of unique 64 nucleotides at 881 to 944 sites in the middle of the sequence which is absent in the type *Nocardia* strains included in the phylogenetic tree. PB-52 differs from *N. niigatensis* IFM 0330 phenotypically where PB-52 possess orange aerial mycelium with light brown vegetative mycelium while *N. niigatensis* IFM 0330 (Kageyama et al., 2004b) produced white aerial mycelium with orange-tan vegetative mycelium. The spore surface of PB-52 was rugose while *N. niigatensis* had smooth spore surface, PB-52 utilized adonitol and maltose while *N. niigatensis* could not. *N. niigatensis* could grow up to a maximum temperature of 37°C while PB-52 grew up to 42°C.

*Nocardia* strain PB-52 showed promising broad spectrum antagonistic activity both in agar medium as well as in culture broth against different bacteria and yeasts. Similar findings were reported by a subset of the researchers (Kavitha et al., 2009, 2010; Mukai et al., 2009). The results signified that the bioactive metabolites having antimicrobial activity were extracellular in nature. Most of the bioactive secondary metabolites including antibiotics produced by actinomycetes are previously reported to be extracellular products (Augustine et al., 2005; Kumar P. S. et al., 2014b). The results of antimicrobial activity of PB-52 strain showed that it secretes broad spectrum antagonistic metabolites which showed the capacity to inhibit the growth of Gram-positive bacteria, Gram-negative bacteria, and *Candida* species. These results are consistent with previous reports of Sun et al. (2007) and Kavitha et al. (2009). The study for evaluation of antimicrobial activity generally involves the search of suitable culture medium. GLM was found to be the best suitable medium for the growth and evaluation of antimicrobial activity of *Nocardia* sp. PB-52 among the tested media. It was obvious from the findings that the antimicrobial activity of PB-52 was positively influenced by the presence of starch as carbon and peptone as the nitrogen source in the medium. This result is quite similar to the previously reported study of El-Gendy et al. (2008) where it was shown that starch was the most suitable carbon source for antibiotic production by *Nocardia* sp. ALAA 2000. It has also been shown that the production of antibiotics in different organisms is strongly influenced by the nature of carbon and nitrogen sources (Vilches et al., 1990). Extraction of the culture broth of PB-52 strain using ethyl acetate led to the recovery of EA-PB-52 consisting of a mixture of metabolites which showed a wide range of activity against the selected test microorganisms. El-Gendy et al. (2008) and Kavitha et al. (2009) have also reported antimicrobial activity of ethyl acetate extracted compound of *Nocardia levis* MK-VL_113 and *Nocardia* sp. ALAA 2000 respectively showing broad spectrum bioactivity.

It is essential to determine the efficiency of the organism during its growth with different culture conditions for determination of the factors that intensify the production of antimicrobial compounds in culture media. PB-52 strain is a mesophilic organism and its capacity of producing antibiotic was consecutively increased with the increase in temperature up to 28°C and pH of 7.4 as well. Complete biosynthesis of antimicrobial compounds production of PB-52 was established on the 8th day of growth. Our result is consistent with the findings of El-Gendy et al. (2008) who reported that *Nocardia* sp. ALAA 2000 showed maximum growth and antibiotic production at 28°C, pH up to 7.4. As reported by Griffiths and Saker (2003), *Cylindrospermopsis raciboskii* produced highest bioactive metabolites when the bacterium moved into the post-exponential phase of growth, while Egorov (1985) suggested that highest antagonistic potential was directly proportional to the value of the biomass. These results signify that cultural environmental aspects like temperature, pH, and incubation period have profound influence on growth and antimicrobial potential of *Nocardia* sp. PB-52 as reported in genus *Nocardia* by El-Gendy et al. (2008).

Non-ribosomal peptides and polyketides are structurally varied group of compounds and have significant biological roles to play. Myriad of bioactive natural products belonging to these two diverse groups have a wide range of applications in the field of medicine, agriculture, and veterinary science (Cane and Walsh, 1999). PCR detection of genes encoding PKS-I and NRPS might be responsible for both of their involvement in the regulation of antimicrobial activity in PB-52. The result is comparable with the studies of Ding et al. (2013) and Passari et al. (2015) where it was clearly shown that few actinomycetes possessing antimicrobial activity were positive for the presence of both of these two biosynthetic pathway genes in their genome. Zhang et al. (2014) also reported that antibacterial activity in *Lysobacter enzymogenes* is regulated by the involvement of both a PKS and NRPS biosynthetic gene. This serves as useful information for discovery of novel bioactive secondary metabolites from PB-52 strain.

MIC of EA-PB-52 at very low concentrations (as low as 0.975 μg/mL) against all the test microorganisms supports the popular notion that *Nocardia* can be one of the best source of potent antimicrobial agents that can be of significance in the treatment of infectious diseases especially those caused by clinically resistant pathogens, such as MRSA, *P. aeruginosa, C. albicans* etc. (Oskay et al., 2004). Our result is comparable with the report of Kumar P. S. et al. (2014b) where the crude ethyl acetate extracted product of *Streptomyces lavendulae* strain SCA5 showed good antimicrobial activity against Gram-positive and Gram-negative bacteria with the MIC-value of 125 μg/mL and the MIC-value against fungi was reported to be 31.25 μg/mL. In addition, Teng_hern et al. (2015) and Ser et al. (2015b) showed that the crude extract of *Streptomyces* sp. MUM256 and *S. pluripotens* MUSC 137 respectively exhibited good antioxidant and anticancer property. The effect of incubating the test microorganisms at 2 × MIC EA-PB-52 lead to rapid decrease in the average log of the viable cells counts whose value was observed to be higher than that treated with 1 × MICs. The considerable decrease in cell counts between 4 and 8 h of incubation period signifies that EA-PB-52 is highly bactericidal/fungicidal seeing that the population of the test microorganisms were almost totally wiped out after 8 h of incubation. Furthermore, the net growth of all the test microorganisms was observed when administered with ½ × MICs of EA-PB-52. Growth inhibition and efficiency of EA-PB-52 was ascertained to be dosage and time-dependent which produces discrete time-kill profiles for the tested microorganisms. Similar antimicrobial activity had also been reported by Olajuyigbe and Afolayan (2012) and Singh et al. (2014). The strong antimicrobial activity of EA-PB-52 was further confirmed by SEM studies which lead to morphological changes in the selected test microorganisms leading to shrinkage and cytosolic loss of the cells. These results are in conformity with the studies of Supaphon et al. (2013) and Nurkanto and Julistiono (2014).

Actinomycetes are known to produce a wide variety of bioactive secondary metabolites possessing diverse biological activity. There are plentiful reports available incorporating the study of GC-MS for chemical analysis of these microbial metabolites (Jog et al., 2014; Teng_hern et al., 2015; Ser et al., 2015a,b). For instance, study by Selvin et al. (2009) illustrated several antimicrobial agents isolated from *Nocardiopsis dassonvillei* MAD08 by using GC-MS. Also, Kim et al. (2008) and Ser et al. (2015a) reported the detection of bioactive compound protocatechualdehyde and an antioxidative agent hexahydro-pyrrolo[1,2-a]pyrazine-1,4-dione in the extract of *S. lincolnensis* M-20 and *S. mangrovisoli* sp. nov., respectively with the help of GC-MS. As such, in this study EA-PB-52 was subjected to GC-MS analysis and twelve chemical compounds were detected with different retention time and abundance. The identified compounds comprised of phenolic compounds, pyrrolizidines, quinones, hydrocarbons, esters, and acids. Phenolic compounds are commonly known as potent antimicrobial agents as well as antioxidant agents as they possess hydrogen-donating capability to reduce free radicals (Yogeswari et al., 2012). Recently, the study conducted by Kumar P. S. et al. (2014c) showed highest antimicrobial activity in the GC-MS fractions containing the highest amount of phenolic compounds. 3,5-bis (1,1-dimethylethyl)-phenol and 2,4-di-t-butyl-6-nitrophenol were the two phenolic compounds detected in EA-PB-52. 3,5-bis (1,1-dimethyethyl)-phenol constituted 34.43% of the total constituents present in EA-PB-52. Antimicrobial activity of 2,4-di-t-butyl-6-nitrophenol is already reported (Kumar P. S. et al., 2014c) but there is no report of 3,5-bis (1,1-dimethyethyl)-phenol as an antimicrobial agent. Roy et al. (2015) reported surfactant activity of 3,5-bis (1,1-dimethyethyl)-phenol in *Nocardiopsis* VITSISB isolated from Marina beach, India. The pyrrolizidine compounds present in EA-PB-52 include hexahydro-pyrrolo[1,2-a]pyrazine-1,4-dione, hexahydro-3-(2-methylpropyl)-pyrrolo[1,2-a]pyrazine-1,4-dione, and hexahydro-3-(phenylmethyl)-pyrrolo[1,2-a]pyrazine-1,4-dione. These compounds had been reported to possess promising antimicrobial activity (Dashti et al., 2014; Manimaran et al., 2015), nematicidal activity (Wang et al., 2014), and antioxidant activity (Ser et al., 2015a). Another study conducted by Devi and Wahab (2012) illustrated that hexahydro-3-(2-methylpropyl)-pyrrolo[1,2-a]pyrazine-1,4-dione in endophytic fungi isolated from *Camellia sinensis* possess strong antimicrobial activity. Furthermore, a quinone compound, 3,5-dihydroxy-4,4-dimethyl-2,5-cyclohexadien-1-one was detected in the PB-52 crude extract for which no antimicrobial activity has been reported till now. Previous study by Sher (2009) demonstrated that the antimicrobial effects of quinines are due to the fact that they are known to complex with nucleophilic amino acids in protein irreversibly. This often leads to loss of function and inactivation of the protein. Asolkar et al. (2010) reported a quinone antibiotic from *Salinispora arenicola* effective against MRSA and other drug-resistant pathogens. Hydrocarbon compounds such as (Z)-3-tetradecene, (E)-5-eicosene, and (E)-9-octadecene are reported to possess antagonistic potential against a wide range of pathogens by a subset of researchers (Cao et al., 2014; Elavarasi et al., 2014; Natarajan and Dhas, 2014). Manilal et al. (2009) reported antibacterial activity of dodecyl acrylate produced by red algae, *Falkenbergia hillebrandii* against multidrug resistant human pathogens. Antimicrobial activity of (Z)-3-tridecene is not available till now. According to the recent reports by Luo et al. (2014), trichloroacetic acid, hexadecyl ester is reported to possess both antioxidant and anticancer activity along with its antimicrobial nature. These compounds are well recognized for their antimicrobial activity and together they may be responsible for the broad spectrum antimicrobial activity of EA-PB-52 against the wide range of test microorganisms. Previous reports by Narayana et al. (2008), Selvin et al. (2009), Ser et al. (2015a,b), and Teng_hern et al. (2015) demonstrated the combinatorial effect of bioactive compounds from GC-MS analysis. Thus, we propose that these compounds could be the key contributing factor for the antimicrobial activities of EA-PB-52. Further, the study of other biological activities of the metabolites produced by the strain PB-52 is the subject of future investigation.

## Conclusion

During the exploration of rare actinomycetes prevailing in forest-derived soil samples of Pobitora Wildlife Sanctuary of Assam, India, *Nocardia* sp. PB-52 was isolated by serial dilution technique. Based on phenotypic and molecular characteristics, the strain was identified as *Nocardia* sp. which shares 99.7% sequence similarity with *N. niigatensis* IFM 0330 (NR_112195). However, the differential phenotypic characteristics on agar media and utilization of carbon sources mainly adonitol and maltose reveal that the strain PB-52 may be classified within the genus *Nocardia* as a different or novel species. Thus to confirm it, further experiments are required such as cellular fatty acid composition, DNA-DNA relatedness value, whole cell sugar analysis etc. of strain PB-52 along with the closest species *N. niigatensis* IFM 0330 and additional type strains.

Extracellular metabolite produced by PB-52 strain exhibited a wide range of antimicrobial activity against Gram-positive bacteria including MRSA, Gram-negative bacteria and yeasts. The antimicrobial potential of *Nocardia* sp. PB-52 was positively influenced by appropriate carbon and nitrogen supplements in the GLM culture media along with the optimum cultural conditions. GLM media constitute of starch and peptone as the principal carbon and nitrogen sources respectively. The maximum production of the antimicrobial compounds was attained on the 8th day of growth at a temperature of 28°C with pH 7.4.

The antimicrobial activity of EA-PB-52 was further confirmed by SEM studies where considerable morphological alterations were observed in the test microbial pathogens. GC-MS analysis revealed that the broad spectrum antimicrobial activity of EA-PB-52 was due to the presence of biologically active compounds. Twelve different compounds were detected in EA-PB-52 which comprised of phenolic compounds, pyrrolizidines, quinones, hydrocarbons, esters, and acids and some of them are already reported to possess antimicrobial or other biological activities. 3,5-bis (1,1-dimethylethyl)-phenol and 2,4-di-t-butyl-6-nitrophenol were the two phenolic compounds detected in EA-PB-52. Phenolic compounds are commonly known as potent antimicrobial agents as well as antioxidant agents. Antimicrobial activity of 2,4-di-t-butyl-6-nitrophenol is already documented but 3,5-bis (1,1-dimethyethyl)-phenol is not reported as an antimicrobial agent. As 3,5-bis (1,1-dimethyethyl)-phenol occupied 34.43% of the total constituents present in EA-PB-52, it might be involved in antimicrobial action.

The genus *Nocardia* was previously reported for the production of antimicrobial compounds against microbial pathogens. Mukai et al. (2009) reported nocardithiocin from *N. pseudobrasiliensis* IFM 0757 active against *Mycobacterium* and *Gordonia* species. Transvalencin A, an antifungal antibiotic is produced from *N. transvalensis* IFM 10065 (Hoshino et al., 2004). Kavitha et al. (2009) reported two bioactive compounds bis-(2-ethylhexyl) phthalate and bis-(5-ethylheptyl) phthalate from *N. levis* MK-VL_113 which showed antagonistic activity against gram-positive bacteria, gram-negative bacteria, yeast, and filamentous fungi. Celmer et al. (1980) reported that *N. argentinensis* produced nargenicin A1 which was found to be active against MRSA. In this work *Nocardia* sp. PB-52 isolated from the soil samples of Pobitora Wildlife Sanctuary, Assam, India exhibited a wide range of antimicrobial activity against Gram-positive bacteria including methicillin resistant *S. aureus* (MRSA), Gram-negative bacteria and yeasts. However, there is no report available regarding the antimicrobial activity of *N. niigatensis* IFM 0330 (NR_112195) which is the closest type strain of PB-52. From our results, it is evident that PB-52 strain could be a promising candidate for the development of potential antimicrobial drug active against a wide range of microbial pathogens including drug resistant microorganisms such as MRSA.

## Author contributions

DT supervised the research work and guided the experimental design. MK provided the research work suggestion. PS conducted the experiments, analyzed the data. DT and PS prepared the manuscript.

### Conflict of interest statement

The authors declare that the research was conducted in the absence of any commercial or financial relationships that could be construed as a potential conflict of interest.
