# Supplementary material for: Broad Spectrum Antimicrobial Activity of Forest-Derived Soil Actinomycete, Nocardia sp. PB-52
Source: Front Microbiol. 2016 Mar 18;7:347. doi: 10.3389/fmicb.2016.00347 (PMC4796592; doi:10.3389/fmicb.2016.00347)
Supplement: Supplementary file 1 [file Table1.PDF]

Table S1: Similarity of PKS-I and NRPS sequences of PB-52 to the closest relatives in GenBank

Biosynthetic gene	Length (bp)	Accession No.	Closest GenBank match (NCBI accession no.)	Identity (%)
PKS-I	1040	KU721843	*Streptomyces olivoviridis* strain O855 clone 22 modular polyketide synthase gene (FJ405974)	89
NRPS	647	KU721842	*Streptomyces* sp. Sp080513GE-23 gene for non-ribosomal peptide synthetase (AB492018)	76
