# Supplementary material for: Broad Spectrum Antimicrobial Activity of Forest-Derived Soil Actinomycete, Nocardia sp. PB-52
Source: Front Microbiol. 2016 Mar 18;7:347. doi: 10.3389/fmicb.2016.00347 (PMC4796592; doi:10.3389/fmicb.2016.00347)
Supplement: Supplementary file 2 [file Presentation1.pptx]

## Slide 1
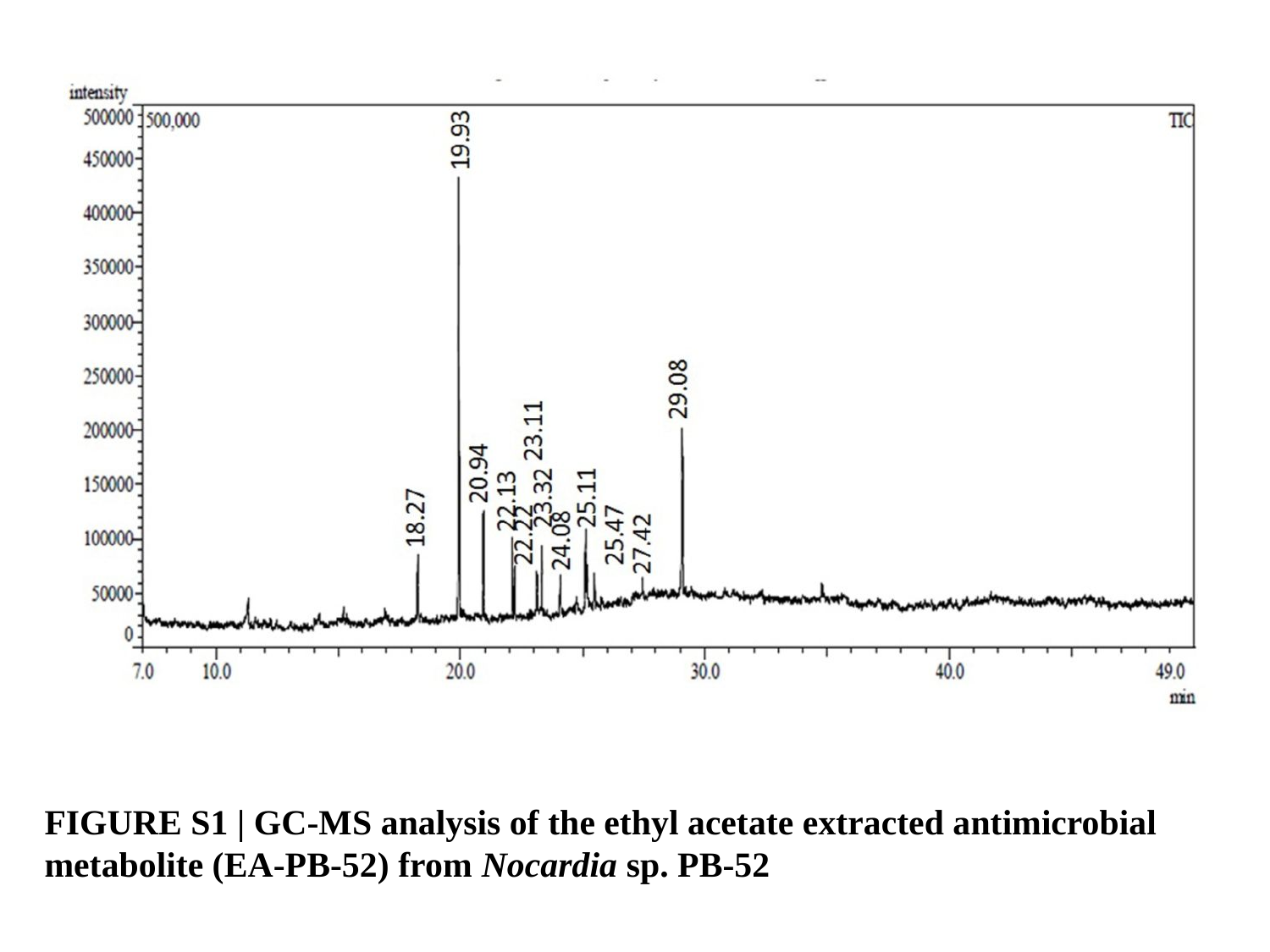

FIGURE S1 | GC-MS analysis of the ethyl acetate extracted antimicrobial metabolite (EA-PB-52) from Nocardia sp. PB-52
