# Supplementary material for: Broad Spectrum Antimicrobial Activity of Forest-Derived Soil Actinomycete, Nocardia sp. PB-52
Source: Front Microbiol. 2016 Mar 18;7:347. doi: 10.3389/fmicb.2016.00347 (PMC4796592; doi:10.3389/fmicb.2016.00347)
Supplement: Supplementary file 3 [file Presentation2.pptx]

## Slide 1
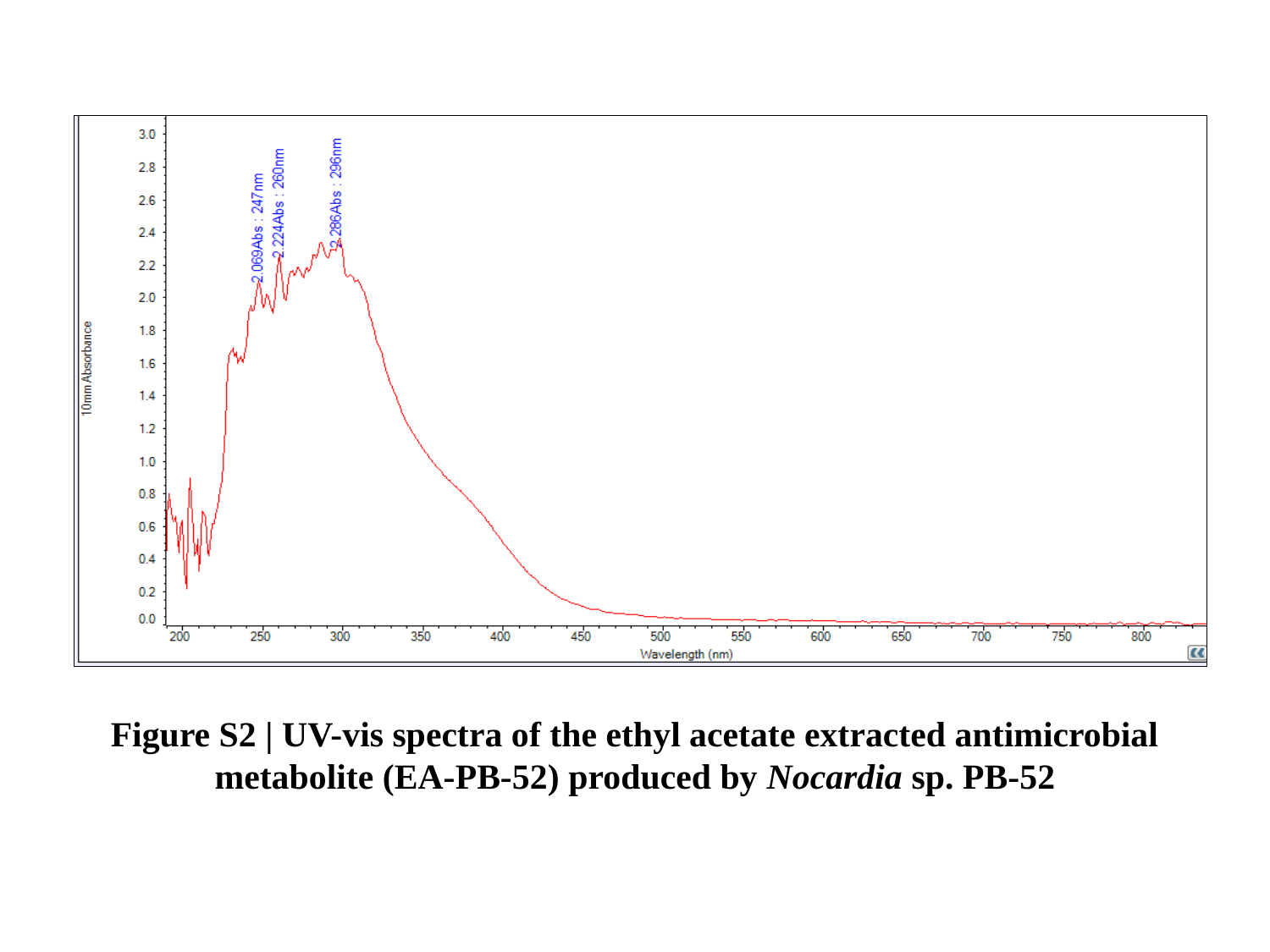

# Figure S2 | UV-vis spectra of the ethyl acetate extracted antimicrobial metabolite (EA-PB-52) produced by Nocardia sp. PB-52
